# Supplementary material for: Cellular Cytotoxicity and Oxidative Potential of Recurrent Molds of the Genus Aspergillus Series Versicolores
Source: Microorganisms. 2022 Jan 20;10(2):228. doi: 10.3390/microorganisms10020228 (PMC8875557; doi:10.3390/microorganisms10020228)
Supplement: Supplementary file 1 [file microorganisms-10-00228-s001.zip › microorganisms-1566847-supplementary.pdf]

*microorganisms*

MDPI

*Article*

# Cellular Cytotoxicity and Oxidative Potential of Recurrent Molds of the Genus *Aspergillus* Series *Versicolores*

**Antoine Géry [1], Charlie Lepetit [1,2], Natacha Heutte [3], Virginie Séguin [1], Julie Bonhomme [1,2] and David Garon [1,*]**

1 ToxEMAC-ABTE, Centre F. Baclesse, Unicaen and Unirouen, Normandie University, 14000 Caen, France; antoine.gery@unicaen.fr (A.G.); charlie.lepetit@gmail.com (C.L.); virginie.seguin@unicaen.fr (V.S.); bonhomme-j@chu-caen.fr (J.B.)
2 Service de Microbiologie, Centre Hospitalier Universitaire de Caen, 14000 Caen, France
3 CETAPS, UFR Sciences et Techniques des Activités Physiques et Sportives Unirouen, Normandie University, 76000 Rouen, France; natacha.heutte@univ-rouen.fr
* Correspondence: david.garon@unicaen.fr

## Supplementary Materials

**Table S1.** *BenA* gene sequences of the isolates used in this study.

| Species | Strain | Sequence |
|---|---|---|
| *Aspergillus amoenus* | CBS245.65 | CgGcgtGGCcAAGAcGAAGTTGTCGGGAcGGAAGAGCTG ACCGAAGGGACCGGCACGGACGGCGTCCATAGTACCG GGCTCGAGATCGACGAGAACGGCACGGGGAACGTACT TGTTGCCGCTGGCCTCGTTGAAGTAGACGTTCATACGCT CGAGCTGGAGGTCGGAGGTACCATTGTAACTGTATTAT ATCAGGGGTTGTGTTCCGTCGTGTTTTGATCGAGTCCTG GACGGGTTGTACTCACACACCGGAGCCATCAAGGCCGT GCTCGCCGGAGATGGTCTGCCTATAGGGTCGTTAGCAA TAAGCACCAAAGGAAATACCATCTGAAATGGATGAAA TTTTCGACGCACCAGAAAGCAGCACCGatatgGTTaCca |
| | HAB06 | CgGacTGGCcAAGacgAAGTTGTCGGGACGGAAGAGCTG ACCGAAGGGACCGGCACGGACGGCGTCCATAGTACCG GGCTCGAGATCGACGAGAACGGCACGGGGAACGTACT TGTTGCCGCTGGCCTCGTTGAAGTAGACGTTCATACGCT CGAGCTGGAGGTCGGAGGTACCATTGTAACTGTATTAT ATCAGGGGTTGTGTTCCGTCGTGTTTTGATCGAGTCCTG GACGGGTTGTACTCACACACCGGAGCCATCAAGGCCGT GCTCGCCGGAGATGGTCTGCCTATAGGGTCGTTAGCAA TAAGCACCAAAGGAAATACCATCTGAAATGGATGAAA TTTTCGACGCACCAGAAAGCAGCACCgatttGGTTaCca |
| *Aspergillus creber* | HOSP150313_5_98 | acGAAGTTGTCGGGAcGGAAAAGCTGACCGAAGGGACC GGCACGGACAGCGTCCATGGTACCGGGCTCGAGATCG ACGAGGACGGCACGAGGAACGTACTTGTTGCCGCTGG CTTCGTTGAAGTAGACGTTCATACGCTCGAGCTGGAGG TCGGAGGTACCATTGTAACTGCATTATATCAGGGGTTGT GTTCTGTCTCGTGTTTTGCTCGAGTCCTGGACGGGTTGT ACTCACACACCGGAGCCATCGAGGCCGTGCTCACCGG AGATGGTCTGCCTATAGAGTCGTTAGCAAAAAAGCACG GAAGGAAATACCATCTGAAATGGATGAAATTTTCGACG CACCAGAAAGCAGCACCGATttGGTTACCa |
| | HOSP050413_5_135 | caagacgAAGTTGTCGGGAcGGAAAAGCTGACCGAAGGG ACCGGCACGGACAGCGTCCATGGTACCGGGCTCGAGA TCGACGAGGACGGCACGAGGAACGTACTTGTTGCCGCT GGCTTCGTTGAAGTAGACGTTCATACGCTCGAGCTGGA GGTCGGAGGTACCATTGTAACTGCATTATATCAGGGGT |

| | | |
|---|---|---|
| | | TGTGTTCTGTCTCGTGTTTTGCTCGAGTCCTGGACGGGTT<br>GTACTCACACACCGGAGCCATCGAGGCCGTGCTCACCG<br>GAGATGGTCTGCCTATAGAGTCGTTAGCAAAAAAGCAC<br>GGAAGGAAATACCATCTGAAATGGATGAAATTTTCGAC<br>GCACCAGAAAGCAGCACCGATTTGGTTACCa |
| | 08FM2_A49 | CgGcgTGGCcAAGAcgAAGTTGTCGGGAcGGAAAAGCTG<br>ACCGAAGGGACCGGCACGGACAGCGTCCATGGTACCG<br>GGCTCGAGATCGACGAGGACGGCACGAGGAACGTACT<br>TGTTGCCGCTGGCCTCGTTGAAGTAGACGTTCATACGCT<br>CGAGTTGGAGGTCGGAGGTACCATTGTAACTGCATTAT<br>ATCAGGGGTTGTGTTCTGTCTCGTGTTTTGCTCGAGTCCT<br>GGACGGGTTGTACTCACACACCGGAGCCATCGAGGCC<br>GTGCTCACCGGAGATGGTCTGCCTATAGAGTCGTTAGC<br>AAAAAAGCACGGAAGGAAATACCATCTGAAATGGATG<br>AAATTTTCGACGCACCAGAAAGCAGCACCGAtttGGTTa<br>CC |
| | HAB02 | tGGCcAAGacgAAGTTGTCGGGAcGGAAAAGCTGACCGA<br>AGGGACCGGCACGGACAGCGTCCATGGTACCGGGCTC<br>GAGATCGACGAGGACGGCACGAGGAACGTACTTGTTG<br>CCGCTGGCTTCGTTGAAGTAGACGTTCATACGCTCGAG<br>CTGGAGGTCGGAGGTACCATTGTAACTGCATTATATCA<br>GGGGTTGTGTTCTGTCTCGTGTTTTGCTCGAGTCCTGGA<br>CGGGTTGTACTCACACACCGGAGCCATCGAGGCCGTGC<br>TCACCGGAGATGGTCTGCCTATAGAGTCGTTAGCAAAA<br>AAGCACGGAAGGAAATACCATCTGAAATGGATGAAAT<br>TTTCGACGCACCAGAAAGCAGCACCgAtttGGTtacc |
| | HAB07 | TGGCcaAGAcGAAGTTGtCGGGAcGGAAAAGCTGACCGA<br>AGGGACCGGCACGGACAGCGTCCATGGTACCGGGCTC<br>GAGATCGACGAGGACGGCACGAGGAACGTACTTGTTG<br>CCGCTGGCTTCGTTGAAGTAGACGTTCATACGCTCGAG<br>CTGGAGGTCGGAGGTACCATTGTAACTGCATTATATCA<br>GGGGTTGTGTTCTGTCTCGTGTTTTGCTCGAGTCCTGGA<br>CGGGTTGTACTCACACACCGGAGCCATCGAGGCCGTGC<br>TCACCGGAGATGGTCTGCCTATAGAGTCGTTAGCAAAA<br>AAGCACGGAAGGAAATACCATCTGAAATGGATGAAAT<br>TTTCGACGCACCAGAAAGCAGCACCGATttGGTTacca |
| | HAB32 | tGGCcAAGacgAAGTTGTCGGGacGGAAAAGCTGACCGA<br>AGGGACCGGCACGGACAGCGTCCATGGTACCGGGCTC<br>GAGATCGACGAGGACGGCACGAGGAACGTACTTGTTG<br>CCGCTGGCTTCGTTGAAGTAGACGTTCATACGCTCGAG<br>CTGGAGGTCGGAGGTACCATTGTAACTGCATTATATCA<br>GGGGTTGTGTTCTGTCTCGTGTTTTGCTCGAGTCCTGGA<br>CGGGTTGTACTCACACACCGGAGCCATCGAGGCCGTGC<br>TCACCGGAGATGGTCTGCCTATAGAGTCGTTAGCAAAA<br>AAGCACGGAAGGAAATACCATCTGAAATGGATGAAAT<br>TTTCGACGCACCAGAAAGCAGCACCGATttGGTTACCa |
| | HAB64 | AcgAAGTTGTCGGGAcGGAAAAGCTGACCGAAGGGACC<br>GGCACGGACAGCGTCCATGGTACCGGGCTCGAGATCG<br>ACGAGGACGGCACGAGGAACGTACTTGTTGCCGCTGG<br>CTTCGTTGAAGTAGACGTTCATACGCTCGAGCTGGAGG<br>TCGGAGGTACCATTGTAACTGCATTATATCAGGGGTTGT<br>GTTCTGTCTCGTGTTTTGCTCGAGTCCTGGACGGGTTGT<br>ACTCACACACCGGAGCCATCGAGGCCGTGCTCACCGG<br>AGATGGTCTGCCTATAGAGTCGTTAGCAAAAAAGCACG<br>GAAGGAAATACCATCtGAaatGgAtgAaATTTtCgACGCAC<br>CagAaAGCAgCaccgAt |

| | | |
|---|---|---|
| *Aspergillus fructus* | 3030204738_C1 | TGGccaagAcgAAGTTGtCGGGaCGGAAGAGCTGAcCGAA<br>GGGACCGGCACGGACAGCGTCCATAGTACCGGGCTCG<br>AGATCGACGAGAACGGCACGGGGAACGTACTTGTTGC<br>CGCTGGCCTCGTTGAAGTAGACGTTCATACGCTCGAGC<br>TGGAGGTCGGAGGTACCATTGTAACTGCATTATATCAG<br>GGGTTGTGTTCGGTCGTGCGTGTTTTGATCGAGTCTTGG<br>ACGGGTTGTACTCACACACCGGAGCCATCAAGGCCGTG<br>CTCGCCGGAGATGGTCTGCCTATAGGGTCGTTAGCAAA<br>AAGCACCAAAGGAAATACCATCTGAAATGGATGAAAT<br>TTTCGACGCACCAGAAAGCAGCACCGATTTGGTTacccac<br>GgT |
| *Aspergillus jensenii* | HAB01 | CgGacTGGCcAaGAcGAAGTTGTCGGGacGGAAAAGCTGA<br>CCGAAGGGACCGGCACGGACAGCGTCCATGGTACCGG<br>GCTCGAGATCGACGAGGACGGCACGAGGAACGTACTT<br>GTTGCCGCTGGCCTCGTTGAAGTAGACGTTCATACGCTC<br>GAGCTGGAGGTCGGAGGTACCATTGTAACTGCATTATA<br>TCAGGGGATGTGTTCTGTCTCGTGTTTTGATCGAGTCCT<br>GGACGGGTTGTACTCACACACCGGAGCCATCGAGGCC<br>GTGCTCACCGGAGATGGTCTGCCTATAGAGTCGTTAGC<br>AAAAAGCACGAAAGGAGATACCATCTGAAATGGATGA<br>AATTTTCGACGCACCAGAAAGCAGCACCGAtttgGTTaCc |
| | 9041799386_C4 | GacTGGCcAAGacgAAGTtGTCGGGAcGGAAAAGCTGACC<br>GAAGGGACCGGCACGGACAGCGTCCATGGTACCGGGC<br>TCGAGATCGACGAGGACGGCACGAGGAACGTACTTGT<br>TGCCGCTGGCCTCGTTGAAGTAGACGTTCATACGCTCG<br>AGCTGGAGGTCGGAGGTACCATTGTAACTGCATTATAT<br>CAGGGGATGTGTTCTGTCTCGTGTTTTGATCGAGTCCTG<br>GACGGGTTGTACTCACACACCGGAGCCATCGAGGCCGT<br>GCTCACCGGAGATGGTCTGCCTATAGAGTCGTTAGCAA<br>AAAGCACGAAAGGAGATACCATCTGAAATGGATGAAA<br>TTTTCGACGCACCAGAAAGCAGCACCGATttGGTTACca |
| | 4070377575_C6 | tGgcaAGacGAAGTTGTCGGGAcGGAAAAGCTGACCGAA<br>GGGACCGGCACGGACAGCGTCCATGGTACCGGGCTCG<br>AGATCGACGAGGACGGCACGAGGAACGTACTTGTTGC<br>CGCTGGCCTCGTTGAAGTAGACGTTCATACGCTCGAGC<br>TGGAGGTCGGAGGTACCATTGTAACTGCATTATATCAG<br>GGGATGTGTTCTGTCTCGTGTTTTGATCGAGTCCTGGAC<br>GGGTTGTACTCACACACCGGAGCCATCGAGGCCGTGCT<br>CACCGGAGATGGTCTGCCTATAGAGTCGTTAGCAAAAA<br>GCACGAAAGGAGATACCATCTGAAATGGATGAAATTTT<br>CGACGCACCAGAAAGCAGCACCGATTTGGTTACCa |
| *Aspergillus protuberus* | HOSP050413_4_129 | tggccAAaGAcgAAGTTGTCGGGAcGGAAGAGCTGACCGA<br>AGGGACCGGCACGGACAGCATCCATAGTACCGGGCTC<br>GAGATCGACGAGAACGGCACGGGGAACGTACTTGTTG<br>CCGCTGGCCTCGTTGAAGTAGACGTTCATACGCTCGAG<br>CTGGAGGTCGGAGGTACCATTGTAACTGCATTATATCA<br>GGGGTTGTGTTCTGTCGTGTTTTGATCGAGTCTTGGACG<br>GGTTGTACTCACACACCGGAGCCATCAAGGCCGTGCTC<br>GCCGGAGATGGTCTGCCTATAGGGTCGTTAGCAAAAAG<br>CACGAAAAGAAATACCATCTGAAATGGATGAAATTTTC<br>GACGCACCAGAAAGCAGCACCGATTTGGTTACCa |
| *Aspergillus puulaauensis* | 0102634450_C10 | ggCcctttcgCacCGGAaTGGCCAAGACGAAGTTGTCGGGAC<br>GGAAGAGCTGACCGAAGGGACCGGCACGGACAGCGTC<br>CATGGTACCGGGCTCGAGATCGACAAGGACGGCACGA<br>GGAACGTACTTGTTGCCGCTGGCCTCGTTGAAGTAGAC<br>GTTCATACGCTCGAGCTGGAGGTCGGAGGTACCATTGT<br>AACTGCATTATATCAGGGGATGTGTTCTGTCTCGTGTTT |

| | | |
|---|---|---|
| | | TGATCGAGTCCTGGACGGGTTGTACTCACACACCGGAG<br>CCATCGAGGCCGTGCTCACCGGAGATGGTCTGCCTATA<br>GAGTCGTTAGCAAAAAGCACGAAAGGAAATACCATCT<br>GAAATGGATGAAATTTTCGACGCACCAGAAAGCAGCA<br>CCGATTTGGTTACCAaa |
| | 4040348777_C2 | tGGCcAAGacgAAGTTGtCGGGacGGAAGAGCTGAcCGAA<br>GGGACCGGCACGGACAGCGTCCATAGTACCAGGCTCG<br>AGATCGACGAGGACGGCACGGGGAACGTACTTGTTGC<br>CGCTGGCCTCGTTGAAGTAGACGTTCATACGCTCGAGC<br>TGGAGGTCGGAGGTACCATTGTAACTGTATTATATCAG<br>GGGCTGTGTTCTGTCGTGTTTTGATCGAGTCCTGGACGG<br>GCTGTACTCACACACCGGAGCCATCGAGGCCGTGCTCA<br>CCGGAGATGGTCTGCCTATAGAGTCGTTAGTAAAAATC<br>ACGAAAACAAAGACCATCTGCAATGGATGAAATTTTC<br>GACGCACCAGAAAGCAGCACCGATTTGGTTaCca |
| | 8051266672_C3 | gGccAAGacgAAGTTGtCGGGacGGAAGAGCTGAcCGAAG<br>GGACCGGCACGGACAGCGTCCATAGTACCAGGCTCGA<br>GATCGACGAGGACGGCACGGGGAACGTACTTGTTGCC<br>GCTGGCCTCGTTGAAGTAGACGTTCATACGCTCGAGCT<br>GGAGGTCGGAGGTACCATTGTAACTGTATTATATCAGG<br>GGCTGTGTTCTGTCGTGTTTTGATCGAGTCCTGGACGGG<br>CTGTACTCACACACCGGAGCCATCGAGGCCGTGCTCAC<br>CGGAGATGGTCTGCCTATAGAGTCGTTAGTAAAAATCA<br>CGAAAGCAAAGACCATCTGCAATGGATGAAATTTTCGA<br>CGCACCAGAAAGCAGCACCGATTTGGTTacc |
| *Aspergillus sydowii* | 9071870945_C5 | AcgAagTTGtCGGGacGGAAGAGCTGACCGAAGGGACCG<br>GCACGGACAGCGTCCATAGTACCAGGCTCGAGATCGA<br>CGAGGACGGCACGGGGAACGTACTTGTTGCCGCTGGCC<br>TCGTTGAAGTAGACGTTCATACGCTCGAGCTGGAGGTC<br>GGAGGTACCATTGTAACTGTATTATATCAGGGGCTGTG<br>TTCTGTCGTGTTTTGATCGAGTCCTGGACGGGCTGTACT<br>CACACACCGGAGCCATCGAGGCCGTGCTCACCGGAGA<br>TGGTCTGCCTATAGAGTCGTTAGTAAAAATCACGAAAG<br>CAAAGACCATCTGCAATGgATGAAATTTTCgACGCACC<br>AgAAAGCAGCACCGATTTGgTTaCCAca |
| | 0062415698_C7 | acgAAGTTGTCGGGacGGAAGAGCTGACCGAAGGGACCG<br>GCACGGACAGCGTCCATAGTACCAGGCTCGAGATCGA<br>CGAGGACGGCACGGGGAACGTACTTGTTGCCGCTGGCC<br>TCGTTGAAGTAGACGTTCATACGCTCGAGCTGGAGGTC<br>GGAGGTACCATTGTAACTGTATTATATCAGGGGCTGTG<br>TTCTGTCGTGTTTTGATCGAGTCCTGGACGGGCTGTACT<br>CACACACCGGAGCCATCGAGGCCGTGCTCACCGGAGA<br>TGGTCTGCCTATAGAGTCGTTAGTAAAAATCACGAAAG<br>CAAAGACCATCTGCAATGGATGAAATTTTCGACGCACC<br>AGAAAGCAGCACCGATTTGGTTACca |
| | 0062445522_C8 | tgGccaAGacgAgTTGTCGGGacGGAAGAGCTGACCGAAGG<br>GACCGGCACGGACAGCGTCCATAGTACCAGGCTCGAG<br>ATCGACGAGGACGGCACGGGGAACGTACTTGTTGCCG<br>CTGGCCTCGTTGAAGTAGACGTTCATACGCTCGAGCTG<br>GAGGTCGGAGGTACCATTGTAACTGTATTATATCAGGG<br>GCTGTGTTCTGTCGTGTTTTGATCGAGTCCTGGACGGGC<br>TGTACTCACACACCGGAGCCATCGAGGCCGTGCTCACC<br>GGAGATGGTCTGCCTATAGAGTCGTTAGTAAAAATCAC<br>GAAAACAAAGACCATCTGCAATGGATGAAATTTTCGA<br>CGCACCAGAAAGCAGCACCGATTTGGTTacca |

| | | |
|---|---|---|
| | 0062445523_C9 | acGAAGTTGTCGGGaCGGAAGAGCTGACCGAAGGGACC<br>GGCACGGACAGCGTCCATAGTACCAGGCTCGAGATCG<br>ACGAGGACGGCACGGGGAACGTACTTGTTGCCGCTGG<br>CCTCGTTGAAGTAGACGTTCATACGCTCGAGCTGGAGG<br>TCGGAGGTACCATTGTAACTGTATTATATCAGGGGCTGT<br>GTTCTGTCGTGTTTTGATCGAGTCCTGGACGGGCTGTAC<br>TCACACACCGGAGCCATCGAGGCCGTGCTCACCGGAG<br>ATGGTCTGCCTATAGAGTCGTTAGTAAAAATCACGAAA<br>ACAAAGACCATCTGCAATGGATGAAATTTTCGACGCAC<br>CAGAAAGCAGCACCGATTTGGTTACc |
| | 0112723999_C11 | TGCCctgtcgcacCGGaacTGGCCAAGACGAAGTTGTCGGGA<br>CGGAAGAGCTGACCGAAGGGACCGGCACGGACAGCGT<br>CCATAGTACCAGGCTCGAGATCGACGAGGACGGCACG<br>GGGAACGTACTTGTTGCCGCTGGCCTCGTTGAAGTAGA<br>CGTTCATACGCTCGAGCTGGAGGTCGGAGGTACCATTG<br>TAACTGTATTATATCAGGGGCTGTGTTCTGTCGTGTTTTG<br>ATCGAGTCCTGGACGGGCTGTACTCACACACCGGAGCC<br>ATCGAGGCCGTGCTCACCGGAGATGGTCTGCCTATAGA<br>GTCGTTAGTAAAAATCACGAAAACAAAGACCATCTGC<br>AATGGATGAAATTTTCGACGCACCAGAAAGCAGCACC<br>GATTTGGTTACcaaatcttttttttgttt |

**List of reagents and sources**

| Reagent/Material | Model/Reference | Provided by |
|---|---|---|
| 5,5'-dithiobis-(2-nitrobenzoic acid) (DTNB) (Ellman's reagent) | 22582 | Thermo Fisher Scientific,Waltham, MA, USA |
| Acetic acid glacial, ReagentPlus®, ≥99% | A6283 | Sigma-Aldrich - Merck, Darmstadt, Germany |
| Bt2a/Bt2b primers | 6422632/6422633 | Eurogentec, Seraing, Belgique |
| Chloramphenicol | 0019 | Cooper, Melun, France |
| Chloroform | ICN19400280 | MP Biomedicals - Thermo Fisher Scientific, Waltham, MA, USA |
| Copper(II) sulfate pentahydrate ACS reagent, ≥98.0% | 209198 | Sigma-Aldrich - Merck, Darmstadt, Germany |
| Cyclonic biocollector | Coriolis® µ | Bertin Technologies, Montigny-le-Bretonneux, France |
| Cytoflex S hemocytometer | B96621 | Beckman Coulter, Brea, CA, USA |
| Dimethyl Sulfoxide (DMSO) | P60-36720100 | Pan Biotech - Dominique Dutscher, Tourgéville, France |
| Dithiotreitol (DTT) | R0861 | Thermo Fisher Scientific,Waltham, MA, USA |
| Dulbecco’s Modified Eagle Medium | 11880028 | Gibco - Thermo Fisher Scientific,Waltham, MA, USA |
| Dulbecco's Phosphate Buffer Solution (10X) | 14200091 | Gibco - Thermo Fisher Scientific,Waltham, MA, USA |
| Ethyl acetate for HPLC, 99.9% | 650528 | Sigma-Aldrich - Merck, Darmstadt, Germany |
| Fetal Bovine Serum, US origin - 100 mL | P30-1401 | Pan Biotech - Dominique Dutscher, Tourgéville, France |
| Gentamicin solution 10 mg/mL | G1272 | Sigma-Aldrich - Merck, Darmstadt, Germany |
| Glycerol | 453755 | Carlo Erba, Val-de-Reuil, France |
| Hybrid Multi-Mode Reader spectrophotometer | Synergy H1 | BioTek - Agilent Technologies, Santa Clara, Californie, États-Unis |
| KOVA™ Glasstic™ Slide 10 | 11917967 | KOVA - Thermo Fisher Scientific, Waltham, MA, USA |
| MALDI-TOF mass spectrometry | Microflex | Bruker, Bremen, Germany |
| NanoDrop 2000 spectrophotometer | ND-2000 | Thermo Fisher Scientific,Waltham, MA, USA |
| NucleoSpin gDNA Clean-up kit | 740230.5 | Macherey-Nagel, Duren, Germany |
| Nucleospin™Plant II kit | 740770.5 | Macherey-Nagel, Duren, Germany |
| Penicilline-streptomycin (10 000 U/ml) | 15140148 | Gibco - Thermo Fisher Scientific,Waltham, MA, USA |
| Proteinase K | V3021 | Promega, Madison, WI, USA |
| PTFE filters - 5 µm porosity | 10068020 | Sartorius - Thermo Fisher Scientific, Waltham, MA, USA |
| Sabouraud dextrose agar with chloramphenicol and gentamicin | 56594 | Bio-Rad, Marnes-la-Coquette, France |
| Sabouraud dextrose agar with chloramphenicol, gentamicin and cycloheximide | 56596 | Bio-Rad, Marnes-la-Coquette, France |
| SpeedVac Plus concentrator | SC210A-230 | Savant - Thermo Fisher Scientific,Waltham, MA, USA |
| Sulforhodamine B | 230162 | Sigma-Aldrich - Merck, Darmstadt, Germany |
| Syringe tip filter - 0.22 µm porosity | CH2225-NP | Thermo Fisher Scientific,Waltham, MA, USA |
| Trichloroacetic acid ACS reagent, ≥99.0% | T6399 | Sigma-Aldrich - Merck, Darmstadt, Germany |
| TRIS base buffer pH 10.5 | T6455 | Sigma-Aldrich - Merck, Darmstadt, Germany |
| Trypsin 0.05% | 11580626 | Gibco - Thermo Fisher Scientific,Waltham, MA, USA |
| Trypsin 0.25% | P10-019100 | Pan Biotech GmbH, Aidenbach, Germany |
| Tween 80 | P4780 | Sigma-Aldrich - Merck, Darmstadt, Germany |
